# Peripheral arterial volume distensibility changes with applied external pressure: significant difference between arteries with different compliance

**DOI:** 10.1038/srep40545

**Published:** 2017-01-17

**Authors:** Mengyan Chen, Aiqing Chen, Xiaoshui Si, Mingxia Ji, Dingchang Zheng

**Affiliations:** 1 Intensive Care Unit, Yiwu Central Hospital, Yiwu City, China; 2 Health and Wellbeing Academy, Faculty of Medical Science, Anglia Ruskin University, Chelmsford, UK

## Abstract

This study aimed to quantify the different effect of external cuff pressure on arterial volume distensibility between peripheral arteries with different compliance. 30 healthy subjects were studied with the arm at two positions (0° and 45° from the horizontal level) to introduce different compliance of arteries. The electrocardiogram and finger and ear photoplethysmograms were recorded simultaneously under five external cuff pressures (0, 10, 20, 30 and 40 mmHg) on the whole arm to obtain arterial volume distensibility. With the applied external cuff pressures of 10, 20, 30 and 40 mmHg, the overall changes in arterial volume distensibility referred to those without external pressure were 0.010, 0.029, 0.054 and 0.108% per mmHg for the arm at the horizontal level, and 0.026, 0.071, 0.170 and 0.389% per mmHg for the arm at 45° from the horizontal level, confirming the non-linearity between arterial volume distensibility and external pressure. More interestingly, the significant differences in arterial volume distensibility changes were observed between the two arm positions, which were 0.016, 0.043, 0.116 and 0.281% per mmHg (all P < 0.01). Our findings demonstrated that arterial volume distensibility of peripheral arm arteries increased with external pressure, with a greater effect for more compliant arteries.

Arteries expand and contract passively and naturally with changes in arterial pulsation. Poor compliance of arteries has been reported as an independent predictor of cardiovascular morbidity^1^,^2^, making the ability to quantify properties of arteries clinically important. Measurement of arterial properties would provide an early marker of risk for cardiovascular diseases, improve patient risk stratification, help in implementing preventive strategies, and also in improving treatment monitoring.

Many terminologies have been used to quantify the properties of arteries, including arterial wall elastic modulus, arterial compliance (volume, lumen area and radius), and arterial distensibility, etc.^3^,^4^,^5^,^6^,^7^. In order to obtain these parameters, ultrasound technique is traditionally used to measure arterial diameter change within each heartbeat or in response to specific conditions including cuff inflation to induce dilation on release^8^,^9^,^10^. Among them, arterial volume distensibility, with the definition of the relative change in blood volume with a known change in arterial transmural pressure (Distensibility = *(∆V/V)/ ∆P*, where *∆V* is the change in blood volume for a segment of artery, and *∆P* is transmural pressure change across the vascular wall, which is the difference between internal vessel pressure and external pressure), is an indirect quantification of the mechanical and hence structural properties of the arterial wall. In real practice, arterial volume distensibility is calculated from the measurement of pulse wave velocity (PWV) or arterial pulse propagation time based on the Bramwell and Hill equation^11^,^12^,^13^ (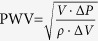, *ρ* is blood density = 1025 kg/m^3^), where the pulse refers to the pulse transmitted down an artery after each heartbeat, from which arterial volume distensibility is derived as:


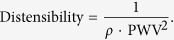


The definition of arterial volume distensibility clearly shows that transmural pressure is one of the most sensitive factors causing the changes of arterial properties. Any changes in internal vessel pressure (the same as the blood pressure) and external pressure on the arteries would alter the properties of arteries. The non-linearity between arterial volume distensibility and blood pressure (BP) has been widely reported^14^,^15^,^16^. As the internal vessel pressure or BP increases, the artery becomes thinner and the ratio of thickness and radius becomes smaller, resulting in lower arterial volume distensibility. Our previous study quantified the inverse and non-linear relationship between arterial volume distensibility and arterial pressure using a simple arm positioning procedure^11^. With the arm positioned above the horizontal level, the arteries are more compliant with higher volume distensibility.

On the other hand, the relationship between arterial properties and external cuff pressure has not been widely investigated using experimental studies. Bank *et al*. reported that elastic properties of the brachial artery change with different transmural pressures induced by an external cuff on the forearm^12^,^17^,^18^,^19^. Recently, the non-linearity between arterial volume distensibility and external pressure has also been experimentally studied by Zheng and Murray^12^. With the increase of external cuff pressure, increased arterial volume distensibility has been observed, with a greater effect at higher external pressure.

However, to the best of our knowledge, there have been no similar experimental studies specifically designed to examine the different effect of external pressure on arterial volume distensibility between peripheral arteries with different compliance or distensibility. This study aimed to provide quantitative evidence on this. The peripheral arm artery with different compliance was induced by raising the arm at different positions. We hypothesized that, with the same external pressure applied on the peripheral arm artery, more compliant the artery is, the bigger change in arterial volume distensibility would be measured.

## Methods

### Subjects

Thirty healthy volunteers (14 male and 16 female) with no history of cardiovascular diseases were recruited, with ages in the range of 23 to 60 years old. There was no age difference between male and female (P = 0.9). Basic subject demographic information including age, height and weight are summarized in Table 1. The propagation distance difference between heart and finger and ear, referred to below as arm length, is also included, which was measured by the length difference between suprasternal notch and tip of index finger and mastoid. The investigation conformed with the principles in the Declaration of Helsinki (World Medical Association 2000). This study was approved by the Local Research Ethics Committee of Yiwu Central Hospital. Informed consent was obtained from all participants in the study.

### Experimental procedure

The study was performed in a quiet clinical measurement room with an ambient temperature of about 23 °C. Subjects were asked to lie supine on a measurement couch for 5 min rest before formal measurements to allow cardiovascular stabilization. photoplethysmography (PPG) probes were attached to the right index fingertip and right earlobe to record finger and ear PPG signals, and ECG electrodes on the body surface for lead-II ECG signal. The PPG signals were measured from the fingertip and earlobe because it has been widely accepted that arterial pulses can be easily detected from the two sites with relatively good and stable signals^20^. A specially designed long cuff (50 cm long) was then wrapped around the whole right arm to introduce external cuff pressure^12^. To ensure the probes and the long cuff were attached with similar tightness for all the subjects, all the measurements in this study were performed by a trained operator.

**Figure 1:**
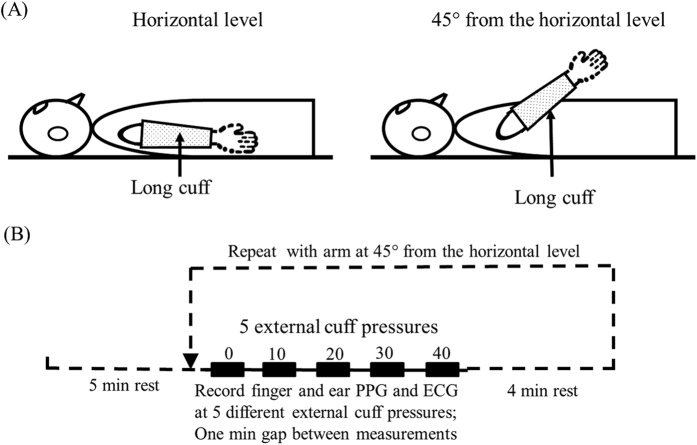
(**A**) Schematic diagram of the arm with a long cuff at two different positions, and (**B**) the experimental procedure.

It has been widely accepted that the arteries are more compliant with lower inner vascular pressure^11^. In this study, as shown in Fig. 1, each subject was asked to position the right arm with long cuff at two different positions (0° and 45° referred to the horizontal level) to induce arteries with different compliances, allowing the effect of external pressure on arterial volume distensibility to be compared between the two arm conditions.

With the arm at each of the two positions (0° and 45° referred to the horizontal level), a series of 5 separate measurements was performed with four different external pressures (0, 10, 20, 30 and 40 mmHg in sequence; they were pressures inflated to the long cuff in reference to the atmospheric pressure), with a total of 10 measurements for each subject. There was a minute rest interval between each measurement. One minute interval was considered to be long enough for the blood vessels to recover back to normal, according to the BP measurement recommendation^21^,^22^ which stated that, when a series of BP readings is taken, the interval between two successive measurements should be at least 1 min. Systolic and diastolic blood pressures (SBP and DBP) were recorded at the beginning and end of all the measurements using a clinically validated automated BP device (Omron M6 Comfort IT) with the arm placed at the horizontal level. Resting mean arterial pressure (MAP) was calculated using the classic formula:


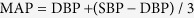


**Figure 2:**
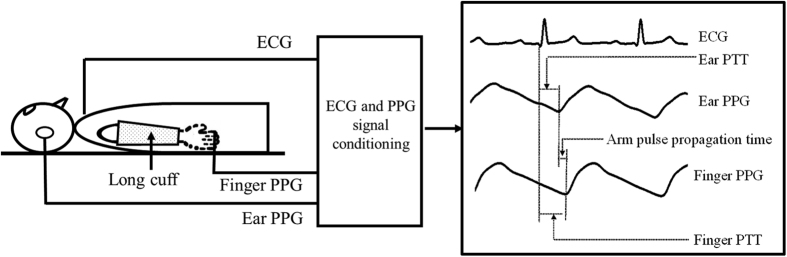
Schematic diagram of the measurement system with illustration of arm pulse propagation time calculation.

For each measurement, after the external cuff pressure was inflated and after stable ECG and finger and ear PPG signals were shown on the display screen of the recording computer, the signals were recorded simultaneously and saved digitally with a sampling rate a sampling rate of 2500 Hz for 120 s for off-line analysis, as shown in Fig. 2. During the signal recording, the arms were kept as still as possible at the required positions, and the subjects were asked to breathe steadily.

Two weeks later, the subjects were invited back to participate in the experiment again, referred to below as a repeat study. The same measurement procedure as in the first study was performed to investigate the between-day measurement repeatability.

### Arm pulse propagation time and arterial volume distensibility calculation

Matlab R2012b (MathWorks Inc.) was used to develop the arterial pulse propagation time and arterial volume distensibility calculation using the method proposed by Zheng and Murray^12^. The whole process is briefly summarized as follows:

The recorded ECG and finger and ear PPG signals were firstly pre-processed using a bandpass filter with a passband from 0.05 to 20 Hz before the identification of the onset of the QRS complex and the corresponding pulse feet of the finger and ear PPGs. As shown in Fig. 2, the beat-by-beat finger and ear PTT were then computed, as well as their difference which was used to represent the arterial pulse propagation time along the major section of the arm, and referred to below as simply arm pulse propagation time (arm pulse propagation time = finger PTT − ear PTT). For each subject, the average of finger PTT, ear PTT and arm pulse propagation time were then calculated from at least 30 reliable beats, which was used as a reference value for that subject. In total, there were 20 values for each of the three parameters (from 5 different external cuff pressures, 2 different arm positions and 2 repeat studies).

Next, the arm pulse wave velocity (PWV) at zero external pressure (PWW_0_) was calculated from: PWV_0_ = arm length/arm pulse propagation time. When the external cuff pressure was applied, PWV for the artery under the cuff was calculated using the equation:


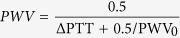


where 0.5 m is cuff length, ΔPTT is arm pulse propagation time change referred to that without external cuff pressure, from which the peripheral arterial volume distensibility was derived as follows:


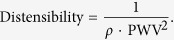


### Calculation of mean transmural pressure across vascular wall

Transmural pressure is the difference between internal arterial pressure and external cuff pressure. According to the linear hydrostatic principle, with the arm at 45° to the horizontal level without applied external cuff pressure, the average decrease of transmural pressure across the vascular wall was equivalent to the internal blood vessel pressure change at the arm mid-point. For instance, with an arm length of 0.66 m, the decrease of transmural pressure could be calculated from (*ρ* gh, where *ρ* is fluid density, g is standard gravity of earth, h is vertical change of the mid-point) as 1025 kg/m^3^ *9.8 m/s^2^* (0.33/1.41) m = 2351 Pa = 18 mmHg. The actual individual transmural pressure changes across vascular wall elicited by positioning the arm at 45° to the horizontal level were then calculated for each subject using individual arm length, separately for the five external cuff pressures.

### Data and statistical analysis

The overall means and SDs of SBP, DBP, MAP, finger PTT, ear PTT, arm pulse propagation time, and arterial volume distensibility were obtained for all subjects, separately for the two different arm positions, five external cuff pressures and two repeat studies. The means and SDs of changes in arm pulse propagation time and arterial volume distensibility induced by the external cuff pressures of 10, 20 30 and 40 mmHg were also obtained. All changes were for paired difference referred to those at zero external pressure in each subject. In addition, the arm pulse propagation time and arterial volume distensibility obtained with the arm at both 0° and 45° from the horizontal level were plotted together against mean transmural pressure across vascular wall, separately for the five external cuff pressures.

SPSS 10.0 software package (SPSS Inc.) was employed to perform variance analysis (ANVOA) to investigate between-day measurement repeatability of the abovementioned parameters and the effect of external cuff pressure and arm position on the changes of arm pulse propagation time and arterial volume distensibility. Post-hoc multiple comparison was used to compare the difference in changes due to the stepped increase in external cuff pressure. All differences were for paired values in each subject, and all statistical analysis was performed on paired data. A value of P < 0.05 was considered statistically significant.

## Results

### Blood pressure

Both SBP and DBP did not demonstrate statistically significant changes during the measurement (both P > 0.1 for SBP and DBP in both studies), which confirmed that the effect of BP changes on arterial pulse propagation times during the measurement was negligible. In comparison with the first study, there was no statistically significant in SBP and DBP from the repeat study (both P > 0.5), although the mean values in the repeat study was slightly lower than that from the first study (111 ± 8 vs 112 ± 8 mmHg for SBP and 68 ± 7 vs 69 ± 8 mmHg for DBP).

### Pulse propagation times with different external cuff pressures

**Figure 3:**
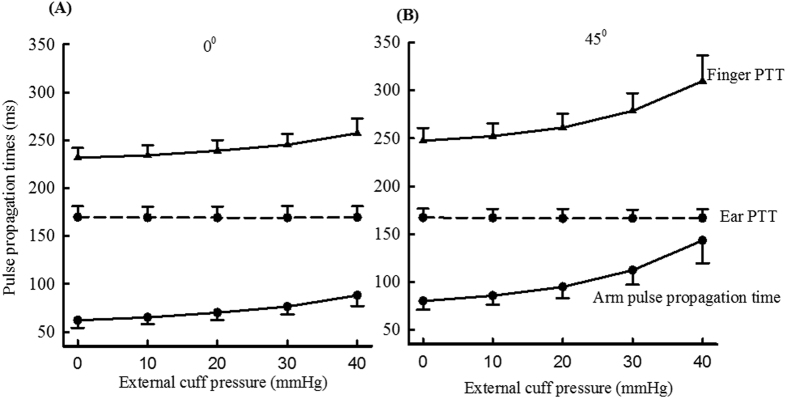
The means and SDs of finger PTT, ear PTT and arm pulse propagation time with different external pressures and with the arm at the horizontal level (**A**) and at 45° from the horizontal level (**B**), which are presented in mean+SD for the finger and ear PTTs, and in mean-SD for the arm pulse propagation time. SDs are population values relating to between-subject variability.

Figure 3 gives the overall means and SDs of PTTs (finger PTT, ear PTT and arm pulse propagation time) with different external pressures (0, 10, 20, 30 and 40 mmHg) and with the arm at 0° and 45° from the horizontal level, calculated from all the data obtained in the first and repeat studies. Variance analysis showed that external cuff pressure caused significant differences in overall measured finger PTT and arm pulse propagation time (both P < 0.001), but not ear PTT. There was no significant difference between the two repeat studies for all the PTTs obtained at certain external cuff pressure and arm position (all P > 0.01). Their average values from the two repeat studies were then used as a reference value for that subject for further analysis.

### Arm pulse propagation time changes with external cuff pressure: difference between measurements from two arm positions

**Figure 4:**
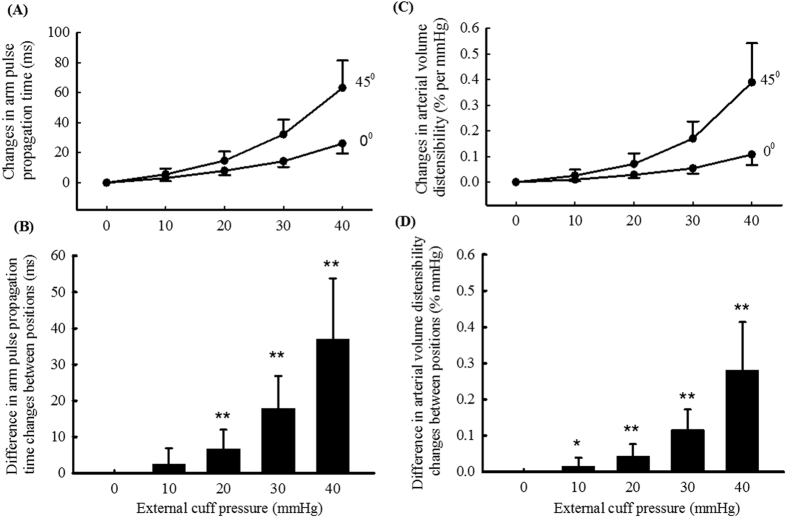
Overall means and SDs of changes in arm pulse propagation time (**A**) and arterial volume distensibility (**B**) with different external pressures, separately for the arm at the horizontal level and at 45° from the horizontal level. Difference in arm pulse propagation time changes (**C**) and arterial volume distensibility changes (**D**) between the two arm positions. *P < 0.05, **P < 0.001.

Figure 4(A) shows the overall means and SDs of arm pulse propagation time changes with external cuff pressures (10, 20, 30 and 40 mmHg) referred to that without external cuff pressure, respectively for the two arm positions. The overall means of arm pulse propagation time changes were 3, 8, 14, 26 with the arm at the horizontal level, and 6, 15, 32, 63 ms with the arm at 45° from the horizontal level. All the changes were significant (all P < 0.001). It is noted that, with the arm at both positions, the changes in arm pulse propagation time were significantly different with the step change of 10 mmHg external cuff pressure (all P < 0.001). With more external cuff pressures, the changes were significantly larger. Therefore, with the increase of external pressure, the changes in arm pulse propagation time increased non-linearly.

Figure 4(B) shows the means and SDs of the differences in arm pulse propagation time changes between the arm positions, where were 2, 7, 18, 37 ms, respectively for the four external cuff pressures (all statistically significant with P < 0.001, except P = 0.02 with the external cuff pressure of 10 mmHg).

### Arterial volume distensibility change with external cuff pressure: difference between measurements from different arm positions

Figure 4(C) shows the overall means and SDs of arterial volume distensibility changes with external cuff pressures (10, 20, 30 and 40 mmHg) referred to those without external cuff pressure, respectively for the two arm positions. The overall mean changes in arterial volume distensibility were 0.010, 0.029, 0.054 and 0.108% per mmHg with the arm at the horizontal level, and 0.026, 0.071, 0.170 and 0.389% per mmHg with the arm at 45° from the horizontal level. Again, with the step increase of 10 mmHg external cuff pressure, arterial volume distensibility changes were significantly different (all P < 0.001), confirming the non-linear relationship between arterial volume distensibility and external cuff pressure at both positions.

Figure 4(D) shows the means and SDs of the differences in arterial volume distensibility changes between the arm positions, which were 0.016, 0.043, 0.116 and 0.281% per mmHg, respectively for the four external pressures (all statistically significant with P < 0.01).

### Non-linear relationship between arterial volume distensibility and vascular transmural pressure

**Figure 5:**
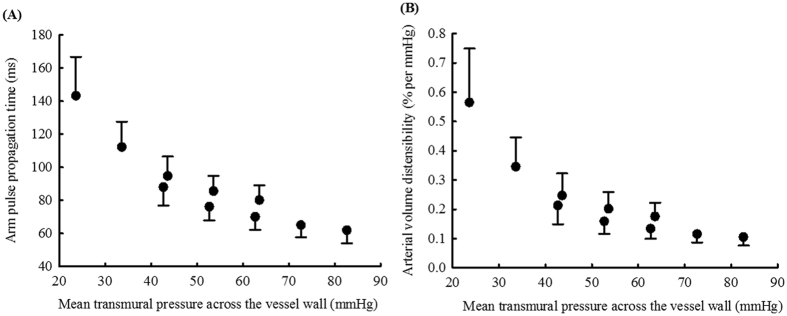
Relationship between mean transmural pressure across vascular wall and arm pulse propagation time (**A**) and arterial volume distensibility (**B**). The data obtained with the arm at the horizontal level (using mean-SD) and at 45° from the horizontal level (using mean+SD) were plotted together.

Figure 5 shows the relationship between mean transmural pressure across the vascular wall and arm pulse propagation time and arterial volume distensibility by combining the data obtained with the arm at the two positions. Their non-linear functions were also observed over a wide range of transmural pressure changes.

### Discussion and Conclusion

In this study, with a long air-filled pressure cuff and by positioning the arm above the horizontal level, the measurement of peripheral arterial volume distensibility using arm propagation time has been performed over a range of low transmural pressures. By positioning the arm at the two different positions (0° and 45° from the horizontal level), when there was no external pressure applied on the arm arteries, the arterial volume distensibility were 0.105% and 0.176% per mmHg, respectively. The difference in baseline distensibility confirmed that the peripheral arm arteries were more compliant when the arm was raised^11^, allowing the different effect of external pressure on arterial volume distensibility between peripheral arteries having different distensibility to be quantified.

This work is an important extension of our previous studies in quantifying arterial volume distensibility changes with transmural pressure. With the arm at horizontal level, the arm pulse propagation time nonlinearly increased by 41% (62 to 88 ms) and arterial volume distensibility by 103% (0.105% to 0.213% per mmHg) with the external cuff pressure from 0 to 40 mmHg. Our measured arm pulse propagation time at zero external pressure (the mean value of 62 ms) agreed with the values from a published study using PPG technique^23^. The changes of arterial volume distensibility with lower transmural pressures were also consistent with our previous study^12^, and agreed with other published studies where different assessment techniques were used and the increased arterial distensibility was reported with lower transmural pressures^18^,^19^,^24^. In this study, the non-linear relationship was also observed with the arm at 45° from the horizontal level, where the arm pulse propagation time nonlinearly increased by 79% (80 to 143 ms), and arterial volume distensibility by 221% (0.176% to 0.565% per mmHg). By combining the data with the arm at both 0° and 45° from the horizontal level, the non-linearity between arterial volume distensibility and different transmural pressures across the vascular wall was observed over a wide range of transmural pressure changes. A future modelling study would be worthwhile to comprehensively investigate different mathematical models to fit the experimental results in order to better understand the mechanical behaviour of arteries with different transmural pressures.

More interestingly, our results quantitatively demonstrated that, with the same external pressure applied on the peripheral arm artery, the more compliant the artery was, the bigger change in arterial volume distensibility was. When the external pressures of 10, 20 30 and 40 mmHg were applied to the peripheral arm arteries, the changes in arterial volume distensibility were all significantly different (with the mean difference of 0.016, 0.043, 0.116 and 0.281% per mmHg) between the two peripheral arm arteries having different compliance. To the best of our knowledge, this is the first experimental study to demonstrate their difference on the peripheral arm arteries with different compliance induced by positioning the arm at two different levels. Our results agreed with the theoretical explanation that, due to the inverse non-linear relationship between arterial volume distensibility and transmural pressure^12^,^25^,^26^,^27^, the pressure effect on distensibility is more pronounced at lower transmural pressures (lower internal vessel pressure or higher external cuff pressure), where the artery is more complaint.

When arterial volume distensibility is measured using traditional techniques under atmospheric condition, the difference between normal and stiffer arteries may not be differentiated. Our findings have demonstrated that, with the same amount of external pressure applied on the arm arteries, arterial volume distensibility of peripheral arm arteries increased more for more compliant arteries. Therefore, when the measurements are performed with external pressures, stiffer arteries with less distensibility changes could be more easily differentiated. This would generate great translational value for arterial volume distensibility measurement using external pressures on the peripheral arm. Our previous study^12^ has already demonstrated that, by applying external pressure on the peripheral arm artery through a long cuff at the horizontal level, the effect of ageing on arterial properties could be easier to be detected when compared with traditional measurement under atmospheric condition. Our findings in this study have implications that there is a great potential to develop a better non-invasive measurement technique by raising the arm at certain levels and simultaneously applying external cuff pressure. When the future technique development moves on to the next step towards specific clinical applications, the measurement protocol should be better considered. Raising the arm to a fixed level could a better option to simplify the measurement procedure.

Nevertheless, we have made an important step in providing scientific support for developing a better arterial distensibility measurement technique, which could be used to easily detect arterial properties changes with different pathophysiological conditions and to monitor therapeutic effectiveness of disease treatment.

## Additional Information

**How to cite this article**: Chen, M. *et al*. Peripheral arterial volume distensibility changes with applied external pressure: significant difference between arteries with different compliance. *Sci. Rep.* **7**, 40545; doi: 10.1038/srep40545 (2017).

**Publisher's note:** Springer Nature remains neutral with regard to jurisdictional claims in published maps and institutional affiliations.

## Figures and Tables

**Table 1:** Demographic data of the subjects in this study.

	Mean ± SD
Age (years)	39 ± 12
Weight (kg)	71 ± 15
Height (cm)	169 ± 9
Arm length (cm)	69 ± 4
